# IL-33-Pretreated Mesenchymal Stem Cells Attenuate Acute Liver Failure by Improving Homing and Polarizing M2 Macrophages

**DOI:** 10.1155/2024/1273099

**Published:** 2024-10-23

**Authors:** Hui Yuan, Yuwen Li, Zihao Kong, Linya Peng, Jiali Song, Xiaoxue Hou, Wen Zhang, Rui Liu, Tiantong Feng, Chuanlong Zhu

**Affiliations:** ^1^Department of Infectious Disease, The First Affiliated Hospital of Nanjing Medical University, Nanjing, China; ^2^Department of Pediatrics, The First Affiliated Hospital of Nanjing Medical University, Nanjing, China; ^3^Department of Gastroenterology, The Affiliated Drum Tower Hospital of Nanjing University Medical School, Nanjing, China; ^4^Department of Infectious and Tropical Diseases, The Second Affiliated Hospital, NHC Key Laboratory of Tropical Disease Control, Hainan Medical University, Haikou, China

**Keywords:** acute liver failure, CCR2/CCL2, interleukin-33, M2 macrophage polarization, mesenchymal stem cells

## Abstract

Mesenchymal stem cells (MSCs) are highly effective in the treatment of acute liver failure (ALF). The efficacy of MSCs is closely related to the inflammatory environment. Therefore, we investigated the functional changes of MSCs in response to interleukin-33 (IL-33) stimulation. The results showed that bone marrow mesenchymal stem cells (BMSCs) pretreated with IL-33 had increased CCR2 expression, targeted CCL2 in the injured liver tissue, and improved the migration ability. Under LPS stimulation, the NF-*κ*B pathway of BMDM was activated, and its phenotype polarized to the M1-type, while BMSCs pretreated with IL-33 inhibited the NF-*κ*B pathway and enhanced M2 macrophage polarization. The M2-type macrophages could further inhibit hepatocytes inflammation, reduce hepatocytes apoptosis, and promote hepatocytes repair. These results suggest that IL-33 can enhance the efficacy of BMSCs in ALF and provide a new strategy for cell therapy of liver diseases.

## 1. Introduction

Hepatotoxic factors, such as hepatitis virus, drugs, and immune damage, can lead to numerous liver cells death, resulting in acute liver failure (ALF). The symptoms of ALF include liver dysfunction, abnormal coagulation, and an altered state of consciousness [1]. Common treatment methods include artificial liver systems and liver transplantation. Artificial liver can promote the elimination and metabolism of toxins in the body, improve hepatic encephalopathy, but cannot enhance survival rate [2, 3]. Liver transplantation is a very effective strategy for treating ALF, but its application is limited by various factors, such as scarce liver sources, poor medical facilities, and graft rejection response [4]. Using mesenchymal stem cells (MSCs) to treat ALF is a new effective strategy that can avoid the limitations of the abovementioned methods.

MSCs are multidirectional stromal cells that can be separated from tissues such as fat, bone marrow, umbilical cord, and amniotic membrane [5]. Due to the lower immunogenicity of MSCs, this greatly reduces the possibility of rejection after transplantation [6]. MSCs are highly migratory. Chemokines, adhesion factors, and the extracellular matrix expressed by MSCs regulate direct homing [7, 8]. Besides regulating tissue repair, MSCs also participate in immune regulation. They regulate many functions by interacting with immune cells. In vivo, MSCs migrate to injured tissue, inhibit inflammation, and promote tissue repair [9].

Many researchers modified MSCs with genes, particles, and chemicals to improve the efficacy. For example, overexpressing c-Met in MSCs can increase their migration ability by targeting hepatocyte growth factor (HGF), which can treat liver injury and significantly improve survival rate [10]. The efficacy of MSCs is also influenced by the inflammatory microenvironment. When MSCs are exposed to low levels of inflammation, their efficacy in promoting repair of injured tissues is reduced, and they might even promote disease progression [11]. Transforming growth factor *β* (TGF-*β*), interleukin-17A, interferon *γ* (IFN-*γ*), and other inflammatory factors, which are important mediators of the inflammatory response, can regulate the immune response [12–14].

A large majority of macrophages found in the human body are liver macrophages, including liver-resident macrophages and infiltrating macrophages [15]. The main members of infiltrating macrophages are bone marrow–derived macrophages (BMDM), which are important to resupply and regeneration after kupffer cells depletion [16]. Studies in recent years have proven that macrophage polarization contributes to the pathology of liver disease [17, 18]. With the different liver diseases and the changes of tissue microenvironment, the role of macrophage polarization also varies [19–21].

Interleukin-33 (IL-33) is a newly discovered cytokine from the IL-1 superfamily that plays an important role in inflammatory and immune responses [22]. It is involved in organ transplantation, tissue repair by increasing the proportion of Th2 and Treg cells [23]. However, few studies [24] have used IL-33 to pretreat bone marrow mesenchymal stem cells (BMSCs). In this study, we found that BMSCs pretreated with IL-33 (BMSCs-33) were more likely to migrate to the damaged liver through the CCR2/CCL2 axis. Subsequently, cytokines secreted by BMSCs-33 promoted the polarization of liver macrophages into M2 macrophages. These M2 macrophages reduced hepatocytes apoptosis and promoted the repair of hepatocytes.

## 2. Materials and Methods

### 2.1. Cell Isolation and Culture

Three-week-old male SD rats were used for the collection of BMSCs. Rats were sacrificed, and then, their femur and tibia were separated. Their bone marrow cavity was rinsed with *α*-MEM. The cells were suspended in 10% FBS and placed in a T25 culture flask after centrifugation at 1000 rpm for 5 min. Then, they were cultured at 37°C and 5% CO_2_ in an incubator. BMSCs-33 were pretreated with 10 ng/mL IL-33 for 24 h. In order to obtain the BMDM, bone marrow was isolated from 8-week-old male SD rats using the same methods described above and then suspended in RPMI-1640 containing 10% FBS along with 10 ng/ml GM-CSF.

### 2.2. Rat Models

SD rats (7-week-old male) were purchased from Beijing Vital River Laboratory Animal Technology company. During the experiment, all rats were kept in a pathogen-free environment with stable temperatures (20−25°C) and humidity (50%–60%). The animals were grouped according to the random number method. In ALF model, D-GalN and LPS were injected intraperitoneally at 850 mg/kg and 10 μg/kg, respectively. All rats were sacrificed using 150 mg/kg sodium pentobarbital.

### 2.3. Flow Cytometry

The phenotypes of BMSCs were identified by flow cytometry (Beckman, USA). BMSCs or BMSCs-33 were incubated with CD29, CD34, CD45, CD90, and MHC II for 30 min. Surface antigen staining has blocked FCR, and isotype controls were made for all stains. All data were analyzed by the FlowJo software. The antibodies used are listed in Table S1.

### 2.4. Western Blotting Assay

The cells or liver tissue were lysed using RIPA buffer (Beyotime Technology, China, P0013B) containing 1 mM PMSF (Beyotime Technology, China, ST506). Equal amounts of proteins were electrophoresed on 10% or 15% SDS-PAGE gels (Epizyme Biotech, China, PG112/PG114) and transferred onto a PVDF membrane. Blocking membranes with 5% bovine serum albumin and incubating them overnight with antibodies at 4°C. These membranes were incubated with HRP-conjugated secondary antibodies for 2 h after three washes with TBST. Finally, the bands were observed using an ECL kit (NCM, China, P10300). The antibodies used in these assays are listed in Table S2.

### 2.5. Reverse Transcription-Polymerase Chain Reaction

Total RNA was extracted using TRIzol (Thermo, USA, 15596–026) and transcribed into cDNA using the Prime Script RT Master Mix (TAKARA, Japan, RR036A). Then, RT-PCR was performed using an ABI Stepone Plus PCR system (Applied Biosystems, USA) and SYBR Green Master Mix (TAKARA, Japan, RR820A). The primer sequences are listed in Table S3.

### 2.6. Transwell Migration Assay

The transwell migration assay was conducted in a transwell chamber with 8-µm pores (Corning, USA, 3422). Serum-starved BMCSs-33 or BMSCs (1 × 10^4^ cells/well) were seeded in the upper chamber, and a serum-free medium containing CCL2 (100 ng/mL; Abcam, USA, ab283927) was added to the lower chamber. After 24 h, the chamber was placed in crystal violet and stained at room temperature for 30 min. Then, the cells were washed with PBS after removing crystal violet. The cells were then dried and photographed under a fluorescence microscope (LEICA, Germany, DMI3000B). The number of cells in each field was recorded.

### 2.7. In Vivo Imaging for Cell Migration

BMSCs in each group were treated with 5 µmol/L DiR (AAT Bioquest, USA) for 20 min and subsequently washed twice with DMEM containing 10% FBS. 1 × 10^6^ cells from each group were injected into ALF rats through the tail veins. After 24 h, the in vivo imaging instrument was performed on BMSCs that had migrated to the liver area (PerkinElmer, USA, IVIS Spectrum Series), and the fluorescence intensity was analyzed by living image.

### 2.8. ELISA

By following the manufacturer's instructions, ELISA kits were used to detect the levels of IL-6, IL-10, and PGE2 in the supernatant of the BMSC culture and also detect the levels of IL-1*β* and IL-6 in the serum of rats. The absorbance was measured at 450 nm with a microplate reader (Thermo, USA).

### 2.9. Histology and Immunohistochemistry

After being fixed in 4% paraformaldehyde for 3 days, the liver tissue was dehydrated and embedded in paraffin, which was later cutted into slices (4 μm thick) and stained with hematoxylin and eosin (H&E). For immunohistochemical staining, the slices were first deparaffinized, rehydrated, and their binding sites were blocked nonspecifically. Then, these slices were incubated in antigen retrieval buffer (Roche) at 37 °C for 30 min and incubated with primary antibodies at 4°C overnight. The following day, these slices were incubated with secondary antibodies for 1 h, exposed to DAB for 5 min, and finally, counterstained with hematoxylin. These slices were scanned and analyzed using image scope software at 40× magnification. The antibodies used in this assay are listed in Table S2.

### 2.10. Immunofluorescence Assay

The cells were fixed with 4% paraformaldehyde for 20 min and permeabilized with 0.5% TritonX-100 for 15 min. Then, the cells were blocked with a blocking buffer (Beyotime Technology, China, P0260) for 30 min and incubated with antibodies at 4°C overnight. Next, the secondary antibodies were added, and the cells were incubated for 1 h at room temperature. Finally, the nuclei were stained with DAPI. The immunofluorescence images were observed under a confocal microscope (Leika, Germany). The antibodies used in this assay are listed in Table S2.

### 2.11. Statistical Analysis

All quantitative experiments were performed in triplicate. The data were statistically analyzed using SPSS 26.0 and expressed as the mean ± standard deviation. Transwell number statistics and the EdU fluorescence ratio analysis were performed using the ImageJ software. The GraphPad Prism 8.0 software was used for mapping. The Shapiro–Wilk test was used to evaluate whether the samples adhere to a normal distribution. In the two-sample comparison, the *t* test was used to analyze the samples conforming to the normal distribution, and the Mann–Whitney *U* test was used to analyze the samples not conforming to the normal distribution. The differences among multiple groups were determined by performing one-way ANOVA, and all differences among and between groups were considered to be statistically significant at *p* < 0.05.

## 3. Results

### 3.1. Comparison of the Inflammatory Cytokines and Chemokine Receptors Between BMSCs and BMSCs-33

**Figure 1:**
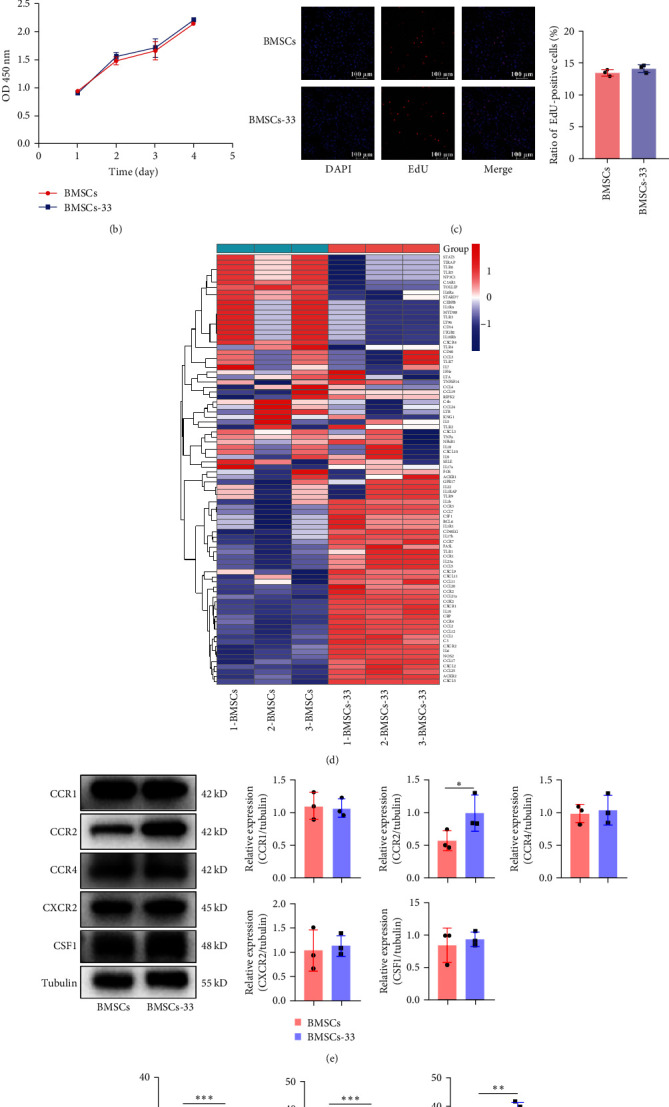
Comparison of inflammatory cytokines and chemokine receptors between BMSCs and BMSCs-33. (A) A flow cytometry assay was performed to identify BMSCs without treatment, and BMSCs pretreated with 10 ng/mL IL-33. CD29 and CD90 were positive, while CD34, CD45, and MHC II were negative. (B) The CCK-8 assay was performed to compare the proliferation ability of the BMSCs group and the BMSCs-33 group. (C) The EdU assay was performed to compare the proliferation ability of the two groups of cells. (D) The difference in the level of cytokines between the BMSCs and BMSCs-33 groups was detected by PCR array. (E) The comparison between the levels of CCR1, CCR2, CCR4, CXCR2, and CSF1 proteins in the BMSCs and BMSCs-33 groups. (F–H) IL-6, IL-10, and PGE2 levels in the cell supernatant of the BMSCs and BMSCs-33 groups were detected by performing ELISA. *⁣*^*∗*^*p* < 0.05, *⁣*^*∗∗*^*p* < 0.01, and *⁣*^*∗∗∗*^*p* < 0.001.

The BMSCs were extracted and cultured to the third generation. In the flow cytometry assay, CD29 and CD90 were positive, while CD34, CD45, and major histocompatibility complex II (MHC II) were negative (Figure 1A). Also, the stem cell characteristics of the BMSCs were preserved after they were pretreated with 10 ng/mL IL-33 for 24 h (Figure 1A). We then compared the proliferation ability between the groups of cells and found there is no difference (Figure 1B,C). To determine whether IL-33 influences the paracrine effect of BMSCs, we compared cytokines expression in the BMSCs and BMSCs-33 groups by PCR array. The results showed that the levels of IL-10, IL-6, CSF1, and COX-2, and chemokine receptors, such as CCR2, CCR4, and CXCR2, increased (Figure 1D). We screened again and found that the levels of CCR2 in chemokine ligands increased significantly (Figure 1E), and the levels of IL-6, IL-10, and PGE2 also increased (Figure1F–H).

### 3.2. BMSCs Pretreated With IL-33 Increased Their Homing to the Damaged Liver Through the CCR2/CCL2 Axis

**Figure 2:**
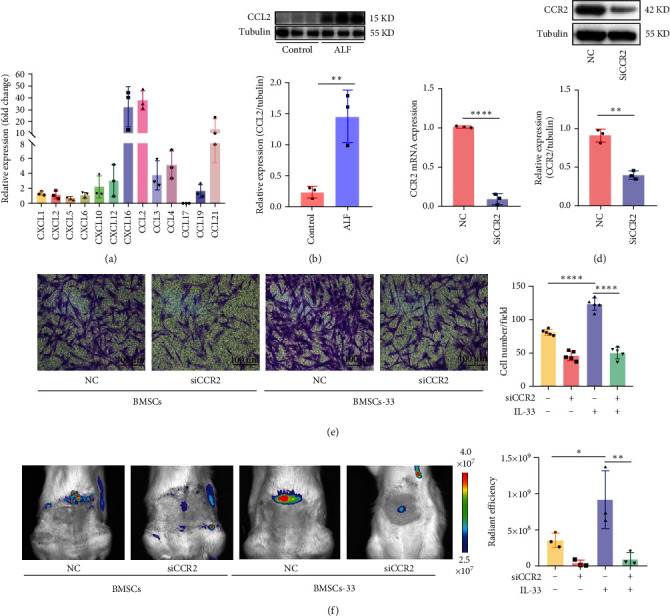
IL-33 increased BMSCs' ability to migrate to the damaged liver through the CCR2/CCL2 axis. (A) Comparison of chemokine ligands in ALF rats' liver. (B) Protein levels of CCL2 in the liver of normal rats and those with ALF. (C, D) The knockdown efficiency of CCR2 in BMSCs was determined. (E) A transwell assay was performed to determine the migration ability of BMSCs to CCL2 in each group. (F) In vivo imaging was performed to observe the homing of BMSCs in rats with ALF. *n* = 3 per group. *⁣*^*∗*^*p* < 0.05, *⁣*^*∗∗*^*p* < 0.01, and *⁣*^*∗∗∗∗*^*p* < 0.0001.

When liver inflammation occurs, it secretes a large number of cytokines, including various chemokines. These chemokines drive immune cells, such as macrophages and lymphocytes, to migrate to liver by binding to corresponding receptors. We found that the expression of CCR2 increased in BMSCs pretreated with IL-33. Therefore, we speculated that the chemokine factor ligands in the liver that bind to CCR2 might increase, which might facilitate BMSCs migrate to liver. To test our hypothesis, we measured the chemokine ligand levels in the liver of rats with ALF and found that CCL2 were significantly elevated (Figure 2A). Further examinations confirmed that CCL2 was significantly elevated in rats with liver failure (Figure 2B). Based on these results, we hypothesized that high levels of CCR2 could bind more effectively to CCL2, which in turn might promote the migration of BMSCs. First, we knocked down the expression of CCR2 in BMSCs and confirmed that the knockdown was successful by performing PCR (Figure 2C) and western blotting assays (Figure 2D). Then, we examined the migration capacity of BMSCs by performing a transwell assay in vitro. We added 100 ng/mL of CCL2 in the lower compartment. The number of migrated cells in the NC-BMSCs, siCCR2-BMSCs, NC-BMSCs-33, and siCCR2-BMSCs-33 was 81.00 ± 4.30, 45.80 ± 7.09, 123.40 ± 9.13, and 50.00 ± 8.63, respectively (Figure 2E). To determine how IL-33 affects the homing ability of BMSCs, we performed tracer experiments in vivo. The BMSCs were stained by DiR in advance to make them fluorescent. According to the results, rats with ALF injected with NC-BMSCs-33 displayed the highest level of fluorescence in their livers. This effect was significantly suppressed after CCR2 was knocked down (Figure 2F). These results showed that IL-33 can increase the expression of CCR2 in BMSCs, and CCR2 can target CCL2 released by damaged hepatocytes, which in turn can improve the homing ability of BMSCs.

### 3.3. BMSCs Pretreated With IL-33 Enhanced the Polarization of M2 Macrophages

**Figure 3:**
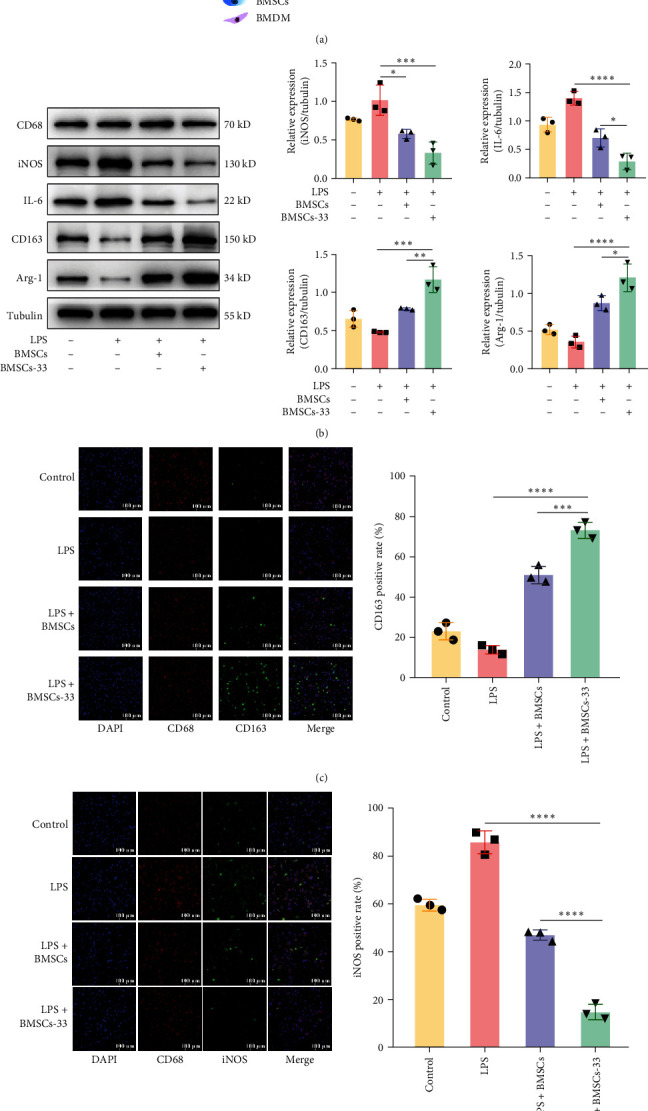
BMSCs pretreated with IL-33 promoted the polarization of M2 macrophages. (A) A coculture pattern diagram with BMDM in the lower chamber and BMSCs in the upper chamber. (B) Western blotting assays were performed to evaluate the expression of iNOS, IL-6, CD163, and Arg–1 in the BMDM of each group. (C, D) The difference in the expression of CD163 and iNOS in each group was compared by immunofluorescence. *⁣*^*∗*^*p* < 0.05, *⁣*^*∗∗*^*p* < 0.01, *⁣*^*∗∗∗*^*p* < 0.001, and *⁣*^*∗∗∗∗*^*p* < 0.0001.

Macrophage is an important type of immune cell in the liver, and its classification is closely associated to disease progression [25]. Our results showed that BMSCs pretreated with IL-33 increased the levels of PGE2, IL-10, and IL-6, which could promote the M2-type polarization of macrophages [26–28]. Therefore, we established a coculture system of BMSCs and macrophages (Figure 3A). Except for the control group, the other three groups were treated with 100 ng/mL LPS for 24 h to polarize M1 macrophage. The BMDM were then cocultured with BMSCs or BMSCs-33 for 48 h. The protein CD68 represented total BMDM, Arg–1 and CD163 were M2-type BMDM markers and iNOS and IL-6 were M1-type BMDM markers [29–31]. The results indicated that Arg–1 and CD163 levels in the LPS group were the lowest. Coculture with BMSCs increased Arg–1 and CD163 levels in BMDM, and BMSCs-33 further increased these changes (Figure 3B). The immunofluorescence assay showed similar results. The fluorescence level of CD163 increased, while the fluorescence level of iNOS and IL-6 decreased significantly in the BMDM that were cocultured with BMSCs-33 (Figure 3C,D).

### 3.4. The NF-*κ*B Pathway Is Crucial for BMSCs-33 to Polarize M2 Macrophages

**Figure 4:**
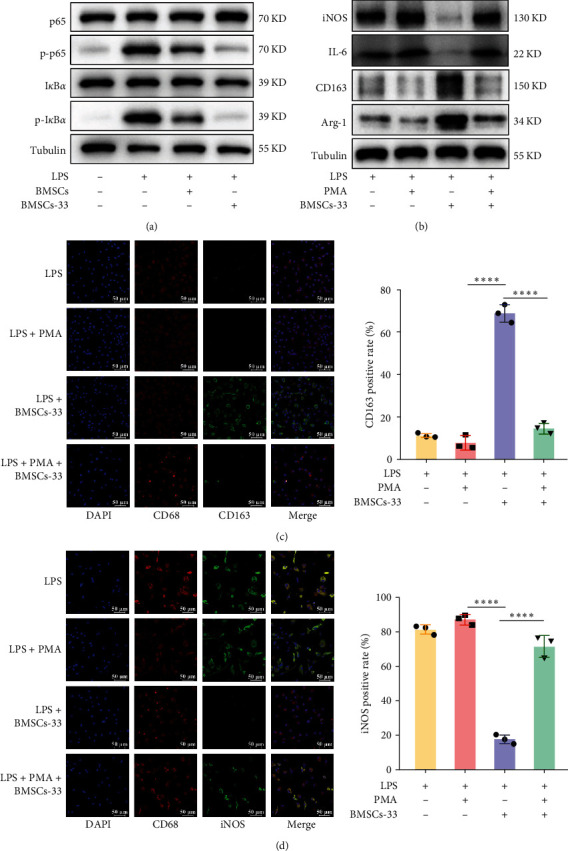
The NF-*κ*B pathway is crucial for BMSCs-33 to regulate M2 macrophage polarization. (A) BMSCs-33 significantly inhibited the expression of p-p65 and p-I*κ*B*α* in BMDM. (B) The expression of CD163 and Arg–1 increased, while the expression of iNOS and IL-6 decreased after BMDM were cocultured with BMSCs-33. This effect was reversed by PMA. (C, D) The differences in the expression of CD163 and iNOS among the groups were compared by immunofluorescence. *⁣*^*∗∗∗∗*^*p* < 0.0001.

In previous studies, toll-like receptor (TLR) 4, which is on the surface of macrophages, was found to be the major receptor for LPS [32, 33]. LPS binds to TLR4 and activates NF-*κ*b through the myd88-dependent pathway, thereby altering the phenotype of macrophages [34]. Therefore, we speculated that BMSCs might affect macrophage polarization through the NF-*κ*B pathway. Our results showed that p-p65 and p-I*κ*B*α* levels in BMDM increased significantly after LPS stimulation, suggesting that the NF-*κ*B pathway was activated. After BMDM were cocultured with BMSCs pretreated with IL-33, p-p65 and p-I*κ*B*α* levels in the BMDM decreased significantly (Figure 4A). To confirm the effect of the NF-*κ*B pathway on macrophage polarization, we applied the NF-*κ*B activator phorbol 12-myristate 13-acetate (PMA) to the BMDM. Under sustained activation, the BMSCs-33-PMA group showed higher protein expression of iNOS and IL-6, as well as lower expression of CD163 and Arg–1 than the BMSCs-33 group (Figure 4B). By performing immunostaining of CD163 and iNOS among different groups, we found that BMSCs-33 significantly increased the fluorescence intensity of CD163, while iNOS fluorescence intensity decreased. However, this effect could be reversed by PMA (Figure 4C,D). These results indicated that BMSCs-33 inhibited the NF-*κ*B pathway of BMDM, thus inducing M2 polarization.

### 3.5. M2 Macrophages Reduced the Apoptosis of the Rat Hepatocyte Line BRL-3A

**Figure 5:**
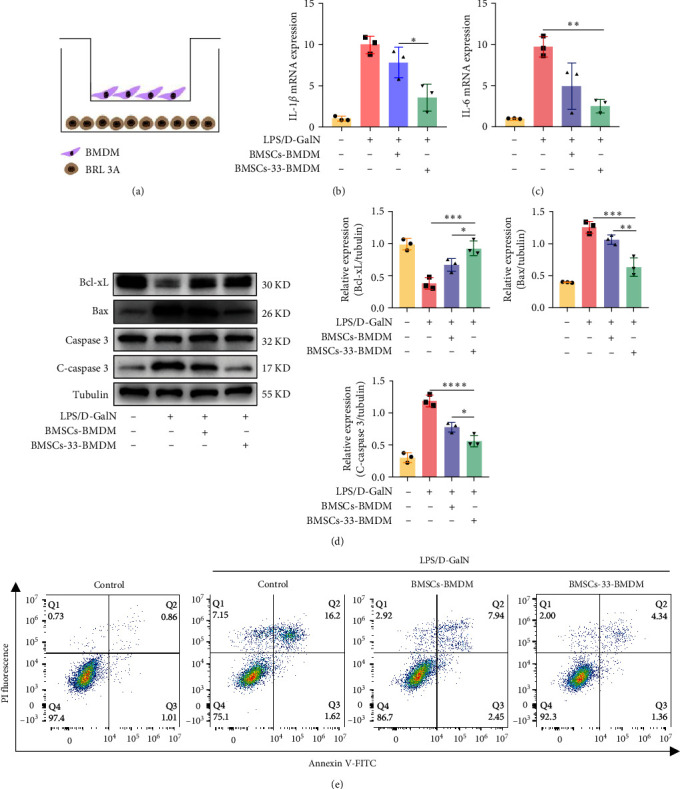
The M2 macrophages decreased the apoptosis of the rat hepatocyte line BRL-3A. (A) A BMDM and BRL-3A cells coculture pattern diagram; BRL-3A cells are in the lower chamber and BMDM are in the upper chamber. (B, C) IL-1*β* and IL-6 mRNA levels in the BRL-3A cells of each group. (D) Western blotting assays were performed to determine the levels of Bax, C-caspase 3, and Bcl-xL in each group. (E) Flow cytometry assays were performed to determine the level of apoptosis in each group. Q2 was the late apoptotic rate, Q3 was the early apoptotic rate. *⁣*^*∗*^*p* < 0.05, *⁣*^*∗∗*^*p* < 0.01, *⁣*^*∗∗∗*^*p* < 0.001, and *⁣*^*∗∗∗∗*^*p* < 0.0001.

To determine whether macrophage polarization can affect the apoptosis of rat hepatocytes, we cocultured BMDM with the rat hepatocyte line BRL-3A (Figure 5A). The control group included normal BRL-3A cells, and the other three groups were treated with 2 mg/mL D-GalN and 1 µg/mL LPS for 24 h, which damaged hepatocytes. The BRL-3A cells were then cocultured with BMDM, where BMDM were cocultured with BMSCs or BMSCs-33 for 48 h in advance. First, we measured the expression of cytokines in each group. When the hepatocytes were damaged, IL-1*β* and IL-6 levels increased significantly. After the BRL-3A cells were cocultured with M2-type macrophages, their level of inflammatory cytokines decreased, and this trend became more prominent as the proportion of M2 increased (Figure 5B,C). Then, the apoptotic protein levels were also measured in four groups. Bax and C-caspase 3 promote apoptosis, while Bcl-xL is anti-apoptotic. We found that Bax and C-caspase 3 in the BMSCs-33-BMDM group decreased significantly, and the level of Bcl-xL increased, which indicated that when hepatocytes were damaged and destroyed, M2-type macrophages decreased further apoptosis (Figure 5D). Finally, flow cytometry assays were performed to detect the late apoptotic cells in Q2. The results demonstrated that the percentage of Q2 cells was lower in the BMSCs-33-BMDM group (Figure 5E).

### 3.6. BMSCs Pretreated With IL-33 Enhanced M2 Macrophage Polarization and Reduced Hepatocyte Apoptosis in Rats With ALF

**Figure 6:**
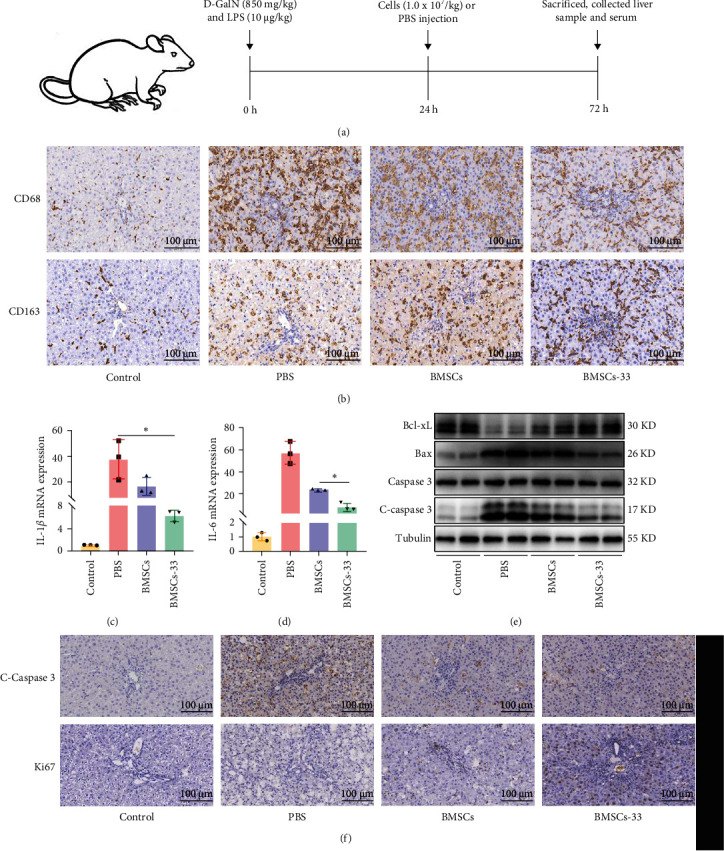
The BMSCs-33 polarized M2 macrophages and decreased the apoptosis of hepatocytes in rats with ALF. (A) Establishment and treatment flowchart of ALF in rats. (B) Immunohistochemistry was used to evaluate CD68 and CD163 levels in the liver macrophages of all groups. The level of CD163 in the BMSCs-33 group was higher than that in the PBS and BMSCs groups. *n* = 3 per group. (C, D) IL-1*β* and IL-6 mRNA levels in the liver tissue of each group. *n* = 3 per group. (E) The levels of the Bax, C-caspase 3, and Bcl-xL proteins in the liver tissue. (F) The expression of C-caspase 3 and Ki67 in the liver tissue of each group was detected by immunohistochemistry. *n* = 3 per group. *⁣*^*∗*^*p* < 0.05.

We performed in vivo experiments to determine the efficacy of BMSCs pretreated with IL-33. The rats were divided into four groups, which included the control group, PBS group, BMSCs group, and BMSCs-33 group, with 10 rats in each group. The rats in the control group were not treated, but those in the other three groups were intraperitoneally injected with 850 mg/kg D-GalN and 10 µg/kg LPS to induce ALF. After 24 h, 1 mL PBS was injected into the tail vein of the rats in the PBS group, and BMSCs or BMSCs-33 were injected into the tail vein of the rats in the BMSCs and BMSCs-33 groups at a dose of 1 × 10^7^ cells/kg (Figure 6A). First, we performed the typing of macrophages in the rat liver by immunohistochemistry (Figure 6B). The CD68-positive rate of rats in the PBS, BMSCs, and BMSCs-33 groups was significantly higher than that in the rats of the control group (Figure 6B). This indicated that a large number of macrophages infiltrated the liver due to inflammation. We compared the positive rate of CD163 in the macrophages of the rats from each group and found that the positive rate of CD163 in the rats of the BMSCs-33 group was higher, while that in the rats of the PBS group was the lowest. This indicated that BMSCs pretreated with IL-33 increased the M2 polarization of intrahepatic macrophages in rats with ALF (Figure 6B). We also compared the levels of inflammatory factors in the liver tissues of each group, and the results showed that BMSCs pretreated with IL-33 significantly reduced IL-1*β* and IL-6 levels in liver (Figure 6C,D). We also measured the apoptotic proteins in the liver tissue and found that the Bcl-xL protein level increased, and the C-caspase 3 and Bax protein levels decreased in the liver tissue of the BMSCs-33 group (Figure 6E). The results of the immunohistochemical analysis revealed that the positive rate of C-caspase 3 decreased and the positive rate of Ki67 increased in the liver of the BMSCs-33 group (Figure 6F). These results indicated that BMSCs pretreated with IL-33 significantly inhibited the apoptosis of hepatocytes and promoted their regeneration.

### 3.7. BMSCs Pretreated With IL-33 Decreased Liver Pathological Scores and Biochemical Indices in Rats With ALF

**Figure 7:**
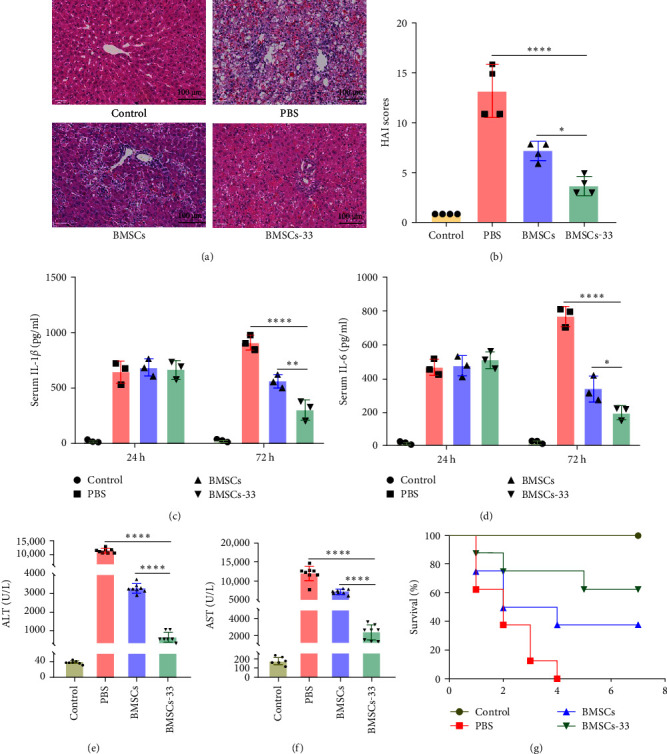
The BMSC-33 decreased the liver pathological scores and biochemical indices in rats with ALF. (A) The liver tissues from different groups were stained by H&E (×400). (B) Histopathological grading of necrosis and inflammation of the liver sections. *n* = 4 per group. (C, D) Serum IL-1*β* and IL-6 levels in the four groups of rats. *n* = 3 per group. (E, F) The levels of ALT and AST in the four groups were detected using a fully automatic biochemical analyzer. *n* = 8 per group. (G) Comparison of the rat survival rates of the four groups after treatment. *⁣*^*∗*^*p* < 0.05, *⁣*^*∗∗*^*p* < 0.01, and *⁣*^*∗∗∗∗*^*p* < 0.0001.

After 48 h of treatment, we collected rat livers for H&E staining. Although the livers in the PBS group showed large areas of necrosis and hepatic cord destruction, those in the BMSCs-33 group only had a small area of hepatocyte spotty necrosis and lesser infiltration of inflammatory cells (Figure 7A). Along with several indicators of hepatic sinusoidal congestion, hepatocyte necrosis, and inflammatory cell infiltration, we estimated the HAI score for liver pathology. The results indicated that the HAI score of BMSCs-33 group was significantly lower than PBS and BMSCs groups (Figure 7B). Then, we analyzed the levels of inflammatory factors and biochemical indicators in rat serum. The results showed that the serum levels of IL-1*β* and IL-6 in PBS group increased with time, while BMSCs and BMSCs-33 could reduce the levels of IL-1*β* and IL-6, and the effect of BMSCs-33 was more significant (*p* < 0.05, Figure 7C,D). In the control group, PBS group, BMSCs group, and BMSCs−33 group, alanine aminotransferase (ALT) levels were 36.33 ± 3.60, 11,669.75 ± 976.93, 3270.82 ± 96.01, and 602.20 ± 246.01 U/L, respectively, and aspartate aminotransferase (AST) levels were 168.17 ± 40.71, 11,966.48 ± 2381.60, 7622.17 ± 216.51, and 2354.48 ±755.30 U/L, respectively. ALT and AST levels in the BMSCs-33 group decreased significantly (*p* < 0.05, Figure 7E,F). Finally, survival rates were determined for each group of rats. All rats in the PBS group died 4 days after treatment, and the 7-day survival rate of the rats in the BMSCs and BMSCs-33 groups was 37.5% and 62.5%, respectively. The BMSCs pretreated with IL-33 significantly increased the survival rate of the rats with ALF (*p* < 0.05, Figure 7G).

## 4. Discussion

The onset of ALF is rapid, and its clinical manifestations include abnormal biochemical indicators, coagulation dysfunction, and even hepatic encephalopathy [35]. ALF patients most commonly die from systemic complications caused by the cytokines storm and damage-related molecular patterns of necrotic hepatocytes, endothelial cells, and white blood cells [36, 37]. MSCs promote hepatocyte regeneration and reduce liver damage; thus, they might be effective in the treatment of ALF. MSCs can differentiate into hepatocytes [38] and also interact with immune cells to regulate the immune capacity of the body [39, 40]. However, MSCs under different inflammatory states show different therapeutic effects. The inefficient migration of BMSCs to the damaged liver also limits their effectiveness. In this study, we found that IL-33 improved the homing and immunosuppression abilities of BMSCs and enhanced the efficacy of BMSCs in ALF rats.

The pro-inflammatory factor IL-33 strongly influences the innate and adaptive immune systems. It acts as an endogenous warning molecule and is usually released by damaged cells [41, 42]. We found that BMSCs pretreated with IL-33 retained their pluripotency and low immunogenicity [13]. Thus, BMSCs pretreated with IL-33 might not cause strong rejection when transplanted in vivo.

Hepatocytes and liver macrophages release high levels of CCL2 in response to liver damage [43]. CCL2, also known as monocyte chemotactic protein 1 (MCP-1), preferentially binds to its receptor CCR2 and recruits immune inhibitory cells [44]. We evaluated the changes in cytokines in BMSCs after pretreatment with IL-33, and the results indicated that CCR2 expression increased significantly (Figure 1E). The binding of CCR2 and CCL2 considerably improved the ability of BMSCs to migrate to damaged hepatocytes. This increased the utilization of BMSCs and enhanced their effectiveness in the damaged liver.

Macrophages are an essential component of innate immunity [45]. They help in maintaining liver homeostasis and promoting acute or chronic liver damage [43]. They limit the potential of host cells to secrete inflammatory mediators by eliminating activated host cells, which is an important mechanism for regulating immunity and reducing indirect injury to host cells and tissue decomposition [46]. Macrophages have different phenotype, including M1 type and M2 type. The M1-type macrophages secrete IL-1*β*, iNOS, TNF-*α*, and other pro-inflammatory factors, while the M2-type macrophages secrete anti-inflammatory components such as IL-10 and TGF-*β* to promote tissue repair [47]. MSCs can polarize M2 macrophages, which can reduce liver inflammation [48, 49]. In this study, BMDM was polarized to the M1 type by LPS, and then, cocultured with BMSCs. The results showed that BMSCs-33 significantly promoted the polarization of BMDM from M1-type to M2-type. This occurred because BMSCs pretreated with IL-33 can secrete more cytokines, such as PGE2, IL-10, and IL-6, which polarize M2 macrophages [15, 50]. Previous studies have shown that PGE2 can also improve ALF by reducing hepatocyte apoptosis and promoting hepatocyte regeneration [49, 51]. As a pro-inflammatory factor, IL-6 is usually increased in response to liver inflammation, and it is generally considered to be a harmful cytokine. However, there is increasing evidence that IL-6 can promote cell proliferation, angiogenesis, and metabolism, which are very important for liver injury [52].

The ubiquitous transcription factor NF-*κ*B is involved in inflammatory regulation and immune response. Normally, the I*κ*B protein masks the nuclear localization signal of NF-*κ*B and prevents it from entering the nucleus [53]. In response to TLR4 and the TNF-*α* receptor (TNFR), I*κ*B is phosphorylated and degraded, followed by the release of the NF-*κ*B dimer, which activates the pathway [54]. Blocking TLR4 and TNFR1 promotes the LPS-induced transformation of M1-type macrophages into M2-type macrophages [55]. Saffron alleviates LPS-induced anxiety and depression by inhibiting the NF-*κ*B pathway and promoting M2-type polarization of macrophages [56]. We found that BMSCs pretreated with IL-33 inhibited the expression of the p-I*κ*B*α* and p-p65 proteins in BMDM (Figure 4A), which in turn led to M2-type polarization of BMDM. To further evaluate the effect of the NF-*κ*B pathway on macrophage polarization, we treated BMDM with the NF-*κ*B activator PMA, which can phosphorylate I*κ*B and thus activate the NF-*κ*B pathway. The results showed that BMSCs-33 alone could induce the polarization of M2 macrophages, but this effect decreased significantly when PMA was added. These findings suggested that the ability of BMSCs-33 to polarize M2 macrophages was strongly inhibited upon sustained activation of the NF-*κ*B pathway. These results indicated that the NF-*κ*B pathway of BMDM was activated via LPS stimulation, and its phenotype polarized toward the M1-type. BMSCs pretreated with IL-33 can inhibit the expression of the NF-*κ*B pathway in BMDM, leading to the M2-type polarization of BMDM.

Almost all hepatocytes in the normal liver are in the G0 phase, but the death and compensatory proliferation of hepatocytes occur when the liver experiences inflammation [57]. In ALF, dead hepatocytes cover a large area, and apoptosis plays a key role in cell death [58]. Thus, inhibiting hepatocyte apoptosis can reduce the damage to liver tissue and decrease the rate of progression of the disease. In this study, BMDM were cocultured with the rat hepatocyte line BRL-3A. The results showed that BMDM cocultured with BMSCs-33 decreased the damage to BRL-3A cells. These cells showed lower mRNA levels of IL-1*β* and IL-6 (Figure 5B,C), lower levels of the C-caspase 3 protein, and higher levels of the Bcl-xL protein (Figure 5D). The results of the flow cytometry assay were consistent with the abovementioned results. Our findings suggested that M2-type BMDM could reduce the apoptosis of injured hepatocytes. In the animal experiments, we found that BMSCs-33 significantly improved the biochemical and prognosis of rats.

Studies on the pretreatment of BMSCs with IL-33 are limited, and the relationship between BMSCs-33 and other immune cells also needs further investigation. Conducting more studies on these aspects can promote the medical application of MSCs.

## 5. Conclusion

To summarize, we found that BMSCs pretreated with IL-33 enhanced the homing ability through the CCR2/CCL2 axis and promoted M2-type polarization by inhibiting the NF-*κ*B pathway of BMDM. The M2-type macrophages inhibited liver inflammation, reduced the apoptosis of hepatocytes, and promoted liver tissue repair (Figure S1). These data provided strategies to improve the homing ability of MSCs to damaged liver tissues for better utilization of the clinical potential of MSCs. Meanwhile, this study showed the immunomodulatory mechanism of BMSCs after IL-33 pretreatment, which can be expected to further develop MSCs-based therapy for liver diseases.

## Data Availability

The data supporting the research result can be obtained from the corresponding author upon reasonable request.

## Nomenclature

MSCs: Mesenchymal stem cells
ALF: Acute liver failure
BMSCs: Bone marrow mesenchymal stem cells
IL-33: Interleukin-33
BMSCs-33: BMSCs pretreated with IL-33
BMDM: Bone marrow–derived macrophages
MHC II: Major histocompatibility complex II
HGF: Hepatocyte growth factor
TGF-*β:*: Transforming growth factor *β*
IFN-*γ*: Interferon *γ*
ALT: Alanine aminotransferase
AST: Aspartate aminotransferase
TLR: Toll-like receptor
MCP-1: Monocyte chemotactic protein 1
TNFR: TNF-*α* receptor.

## Supporting Information

Supporting Information Table S1. Antibodies for flow cytometry analysis. Table S2. Primary and secondary antibodies. Table S3. Sequence (5′ to 3′) of specific primers used for RT-PCR analysis. Figure S1. Schematic representation of BMSCs-33 to attenuate hepatocyte damage in rats with ALF. The expression of CCR2 in BMSCs pretreated with IL-33 increased, and it combined with CCL2 secreted by hepatocytes which could promote the migration of BMSCs. BMSCs-33 secreted IL-10, PGE2, and IL-6, which improved the inflammatory microenvironment. They inhibited the NF-*κ*B pathway of macrophages, thereby promoting macrophage M2 polarization. Finally, M2 macrophages could reduce hepatocytes apoptosis.

## References

[1] Wendon J., Cordoba J., Dhawan A. (2017). EASL Clinical Practical Guidelines on the Management of Acute (Fulminant) Liver Failure. *Journal of Hepatology*.

[2] Tsipotis E., Shuja A., Jaber B. L. (2015). Albumin Dialysis for Liver Failure: A Systematic Review. *Advances in Chronic Kidney Disease*.

[3] Stutchfield B. M., Simpson K., Wigmore S. J. (2011). Systematic Review and Meta-Analysis of Survival following Extracorporeal Liver Support. *British Journal of Surgery*.

[4] Olivo R., Guarrera J. V., Pyrsopoulos N. T. (2018). Liver Transplantation for Acute Liver Failure. *Clinics in Liver Disease*.

[5] Natsumeda M., Florea V., Rieger A. C. (2017). A Combination of Allogeneic Stem Cells Promotes Cardiac Regeneration. *Journal of the American College of Cardiology*.

[6] Klyushnenkova E., Mosca J. D., Zernetkina V. (2005). T Cell Responses to Allogeneic Human Mesenchymal Stem Cells: Immunogenicity, Tolerance, and Suppression. *Journal of Biomedical Science*.

[7] Kucia M., Reca R., Miekus K. (2005). Trafficking of Normal Stem Cells and Metastasis of Cancer Stem Cells Involve Similar Mechanisms: Pivotal Role of the SDF-1–CXCR4 Axis. *Stem Cells*.

[8] Chute J. P. (2006). Stem Cell Homing. *Current Opinion in Hematology*.

[9] Uccelli A., Moretta L., Pistoia V. (2008). Mesenchymal Stem Cells in Health and Disease. *Nature Reviews Immunology*.

[10] Wang K., Li Y., Zhu T. (2017). Overexpression of c-Met in Bone Marrow Mesenchymal Stem Cells Improves Their Effectiveness in Homing and Repair of Acute Liver Failure. *Stem Cell Research & Therapy*.

[11] Wang Y., Chen X., Cao W., Shi Y. (2014). Plasticity of Mesenchymal Stem Cells in Immunomodulation: Pathological and Therapeutic Implications. *Nature Immunology*.

[12] Xu C., Yu P., Han X. (2014). TGF-*β* Promotes Immune Responses in the Presence of Mesenchymal Stem Cells. *The Journal of Immunology*.

[13] Sivanathan K. N., Rojas-Canales D. M., Hope C. M. (2015). Interleukin-17A-Induced Human Mesenchymal Stem Cells Are Superior Modulators of Immunological Function. *Stem Cells*.

[14] Kim D. S., Jang I. K., Lee M. W. (2018). Enhanced Immunosuppressive Properties of Human Mesenchymal Stem Cells Primed by Interferon-Gamma. *EBioMedicine*.

[15] Wang C., Ma C., Gong L. (2021). Macrophage Polarization and Its Role in Liver Disease. *Frontiers in Immunology*.

[16] Guillot A., Tacke F. (2019). Liver Macrophages: Old Dogmas and New Insights. *Hepatology Communications*.

[17] Liu J., Zhang S., Cao H. (2017). Deficiency of p38*α* in Macrophage Ameliorates d-Galactosamine/TNF-*α*-Induced Acute Liver Injury in Mice. *The FEBS Journal*.

[18] Peng J., Li J., Huang J. (2019). P300/CBP Inhibitor A-485 Alleviates Acute Liver Injury by Regulating Macrophage Activation and Polarization. *Theranostics*.

[19] Ma P.-F., Gao C.-C., Yi J. (2017). Cytotherapy with M1-Polarized Macrophages Ameliorates Liver Fibrosis by Modulating Immune Microenvironment in Mice. *Journal of Hepatology*.

[20] Yang Y., Ye Y.-C., Chen Y. (2018). Crosstalk Between Hepatic Tumor Cells and Macrophages via Wnt/*β*-Catenin Signaling Promotes M2-Like Macrophage Polarization and Reinforces Tumor malignant Behaviors. *Cell Death & Disease*.

[21] Kazankov K., Jørgensen S. M. D., Thomsen K. L. (2019). The Role of Macrophages in Nonalcoholic Fatty Liver Disease and Nonalcoholic Steatohepatitis. *Nature Reviews Gastroenterology & Hepatology*.

[22] Schmitz J., Owyang A., Oldham E. (2005). IL-33, an Interleukin-1-Like Cytokine that Signals via the IL-1 Receptor-Related Protein ST2 and Induces T Helper Type 2-Associated Cytokines [J]. *Immunity*.

[23] Liu Q., Turnquist H. R. (2016). Controlling the Burn and Fueling the Fire: Defining the Role for the Alarmin Interleukin-33 in Alloimmunity. *Current Opinion in Organ Transplantation*.

[24] Terraza C., Fuentes R., Pino-Lagos K. (2018). IFN-*γ* and IL-33 Modulate Mesenchymal Stem Cells Function Targeting Th1/Th17 Axis in a Murine Skin Transplantation Model. *Cytokine*.

[25] Li M., Sun X., Zhao J. (2020). CCL5 Deficiency Promotes Liver Repair by Improving Inflammation Resolution and Liver Regeneration Through M2 Macrophage Polarization. *Cellular & Molecular Immunology*.

[26] Cao X., Duan L., Hou H. (2020). IGF-1C Hydrogel Improves the Therapeutic Effects of MSCs on Colitis in Mice through PGE_2_-Mediated M2 Macrophage Polarization. *Theranostics*.

[27] Jung M., Ma Y., Iyer R. P. (2017). IL-10 Improves Cardiac Remodeling After Myocardial Infarction by Stimulating M2 Macrophage Polarization and Fibroblast Activation. *Basic Research in Cardiology*.

[28] Dichtl S., Lindenthal L., Zeitler L. (2021). Lactate and IL6 Define Separable Paths of Inflammatory Metabolic Adaptation. *Science Advances*.

[29] Mossanen J. C., Krenkel O., Ergen C. (2016). Chemokine (C-C Motif) Receptor 2-Positive Monocytes Aggravate the Early Phase of Acetaminophen-Induced Acute Liver Injury. *Hepatology*.

[30] Liu K., Zhao E., Ilyas G. (2015). Impaired Macrophage Autophagy Increases the Immune Response in Obese Mice by Promoting Proinflammatory Macrophage Polarization. *Autophagy*.

[31] Cho D.-I., Kim M. R., Jeong H.-Y. (2014). Mesenchymal Stem Cells Reciprocally Regulate the M1/M2 Balance in Mouse Bone Marrow-Derived Macrophages. *Experimental & Molecular Medicine*.

[32] Geng J., Shi Y., Zhang J. (2021). TLR4 Signalling via Piezo1 Engages and Enhances the Macrophage Mediated Host Response During Bacterial Infection. *Nature Communications*.

[33] Ciesielska A., Matyjek M., Kwiatkowska K. (2021). TLR4 and CD14 Trafficking and Its Influence on LPS-Induced pro-Inflammatory Signaling. *Cellular and Molecular Life Sciences*.

[34] Chen X.-X., Tang L., Fu Y.-M., Wang Y., Han Z.-H., Meng J.-G. (2017). Paralemmin-3 Contributes to Lipopolysaccharide-Induced Inflammatory Response and Is Involved in Lipopolysaccharide-Toll-Like Receptor-4 Signaling in Alveolar Macrophages. *International Journal of Molecular Medicine*.

[35] Bernal W., Wendon J. (2013). Acute Liver Failure. *New England Journal of Medicine*.

[36] Jalan R., Pollok A., Shah S. H. A., Madhavan K. K., Simpson K. J. (2002). Liver Derived pro-Inflammatory Cytokines May be Important in Producing Intracranial Hypertension in Acute Liver Failure. *Journal of Hepatology*.

[37] Chung R. T., Stravitz R. T., Fontana R. J. (2012). Pathogenesis of Liver Injury in Acute Liver Failure. *Gastroenterology*.

[38] Lee K.-D., Kuo T. K.-C., Whang-Peng J. (2004). In Vitro Hepatic Differentiation of Human Mesenchymal Stem Cells. *Hepatology*.

[39] Shi M., Liu Z.-W., Wang F.-S. (2011). Immunomodulatory Properties and Therapeutic Application of Mesenchymal Stem Cells. *Clinical and Experimental Immunology*.

[40] Singer N. G., Caplan A. I. (2011). Mesenchymal Stem Cells: Mechanisms of Inflammation. *Annual Review of Pathology: Mechanisms of Disease*.

[41] Liew F. Y., Girard J.-P., Turnquist H. R. (2016). Interleukin-33 in Health and Disease. *Nature Reviews Immunology*.

[42] Mantovani A., Dinarello C. A., Molgora M., Garlanda C. (2019). Interleukin-1 and Related Cytokines in the Regulation of Inflammation and Immunity. *Immunity*.

[43] Ju C., Tacke F. (2016). Hepatic Macrophages in Homeostasis and Liver Diseases: From Pathogenesis to Novel Therapeutic Strategies. *Cellular & Molecular Immunology*.

[44] Xu M., Wang Y., Xia R., Wei Y., Wei X. (2021). Role of the CCL2-CCR2 Signalling Axis in Cancer: Mechanisms and Therapeutic Targeting. *Cell Proliferation*.

[45] Sica A., Mantovani A. (2012). Macrophage Plasticity and Polarization: In Vivo Veritas. *Journal of Clinical Investigation*.

[46] Bilzer M., Roggel F., Gerbes A. L. (2006). Role of Kupffer Cells in Host Defense and Liver Disease. *Liver International*.

[47] Wynn T. A., Chawla A., Pollard J. W. (2013). Macrophage Biology in Development, Homeostasis and Disease. *Nature*.

[48] Li Y., Sheng Q., Zhang C. (2021). STAT6 up-Regulation Amplifies M2 Macrophage Anti-Inflammatory Capacity through Mesenchymal Stem Cells. *International Immunopharmacology*.

[49] Wang J., Liu Y., Ding H., Shi X., Ren H. (2021). Mesenchymal Stem Cell-Secreted Prostaglandin E2 Ameliorates Acute Liver Failure via Attenuation of Cell Death and Regulation of Macrophage Polarization. *Stem Cell Research & Therapy*.

[50] Xu J., Zhang J., Zhang Z. (2021). Hypoxic Glioma-Derived Exosomes Promote M2-Like Macrophage Polarization by Enhancing Autophagy Induction. *Cell Death & Disease*.

[51] Liu Y., Ren H., Wang J. (2019). Prostaglandin E_2_ Secreted by Mesenchymal Stem Cells Protects Against Acute Liver Failure *via* Enhancing Hepatocyte Proliferation. *The FASEB Journal*.

[52] Naseem S., Hussain T., Manzoor S. (2018). Interleukin-6: A Promising Cytokine to Support Liver Regeneration and Adaptive Immunity in Liver Pathologies. *Cytokine & Growth Factor Reviews*.

[53] Karin M. (1999). How NF-*κ*B is Activated: the Role of the I*κ*B Kinase (IKK) Complex. *Oncogene*.

[54] Oeckinghaus A., Ghosh S. (2009). The NF-*κ*B Family of Transcription Factors and Its Regulation. *Cold Spring Harbor Perspectives in Biology*.

[55] Sawoo R., Dey R., Ghosh R., Bishayi B. (2021). TLR4 and TNFR1 Blockade Dampen M1 Macrophage Activation and Shifts Them towards an M2 Phenotype. *Immunologic Research*.

[56] Zhang L., Previn R., Lu L., Liao R.-F., Jin Y., Wang R.-K. (2018). Crocin, a Natural Product Attenuates Lipopolysaccharide-Induced Anxiety and Depressive-Like Behaviors through Suppressing NF-*k*B and NLRP3 Signaling Pathway. *Brain Research Bulletin*.

[57] Schwabe R. F., Luedde T. (2018). Apoptosis and Necroptosis in the Liver: A Matter of Life and Death. *Nature Reviews Gastroenterology & Hepatology*.

[58] Galle P. R., Hofmann W. J., Walczak H. (1995). Involvement of the CD95 (APO-1/Fas) Receptor and Ligand in Liver Damage. *The Journal of Experimental Medicine*.

