# Supplementary material for: IL-33-Pretreated Mesenchymal Stem Cells Attenuate Acute Liver Failure by Improving Homing and Polarizing M2 Macrophages
Source: Stem Cells Int. 2024 Oct 23;2024:1273099. doi: 10.1155/2024/1273099 (PMC11524710; doi:10.1155/2024/1273099)
Supplement: Supporting Information — Table S1. Antibodies for flow cytometry analysis. Table S2. Primary and secondary antibodies. Table S3. Sequence (5′ to 3′) of specific primers used for RT-PCR analysis. Figure S1. Schematic representation of BMSCs-33 to attenuate hepatocyte damage in rats with ALF. The expression of CCR2 in BMSCs pretreated with IL-33 increased, and it combined with CCL2 secreted by hepatocytes which could promote the migration of BMSCs. BMSCs-33 secreted IL-10, PGE2, and IL-6, which improved the inflammatory microenvironment. They inhibited the NF-κB pathway of macrophages, thereby promoting macrophage M2 polarization. Finally, M2 macrophages could reduce hepatocytes apoptosis. [file 1273099.f1.docx]

**Supplementary materials**

**Table S1. Antibodies for flow cytometry analysis**

| Product | Catalogue Number | Supplier |
| --- | --- | --- |
| FITC anti-rat CD29 | 102205 | BioLegend |
| anti- rat CD34 | PA5-85917 | Thermos fisher scientific |
| PE anti- rat CD45 | 202207 | BioLegend |
| PE anti- rat CD90 | 202523 | BioLegend |
| PE anti- rat MHCⅡ | 12-5322-81 | ebioscience |

**Table S2. Primary and secondary antibodies**

| Product | Catalogue Number | Supplier |
| --- | --- | --- |
| WB:  Primary antibodies:  mouse anti-rat Tubulin  rabbit anti-rat Caspase3 | M20023  14220 | Abmart  Cell Signaling Technology |
| rabbit anti-rat C-Caspase3 | 9664 | Cell Signaling Technology |
| rabbit anti-rat Bcl-xL  rabbit anti-rat Bax | 2764  50599 | Cell Signaling Technology  proteintech |
| rabbit anti-rat CCR2 | YT5635 | ImmunoWay |
| rabbit anti-rat CSF1 | YT5553 | ImmunoWay |
| rabbit anti-rat CXCR2 | YT5397 | ImmunoWay |
| mouse anti-rat CCR1 | Bs-1169R | Bioss |
| mouse anti-rat CCR4 | Bs-20788R | Bioss |
| rabbit anti-rat CD163 | Ab82422 | Abcam |
| rabbit anti-rat CD68 | Ab283654 | Abcam |
| rabbit anti-rat iNOS  rabbit anti-rat p65  rabbit anti-rat p-p65  rabbit anti-rat IκBα  rabbit anti-rat p-IκBα | Ab178945  4764  3033  4812  2859 | Abcam  Cell Signaling Technology  Cell Signaling Technology  Cell Signaling Technology  Cell Signaling Technology |
| Secondary antibodies: |  |  |
| anti-rabbit IgG HRP-linked Ab | AS014 | ABclonal |
| anti-mouse IgG HRP-linked Ab | AS003 | ABclonal |
| IF:  Primary antibody:  rabbit anti-rat CD163  mouse anti-rat CD68  Secondary antibody:  Alexa Fluor 488 goat anti-rabbit IgG  Alexa Fluor 647 goat anti-mouse IgG  IHC: | Ab82422  Sc-20060  Ab150077  A0473 | Abcam  Santa cruz  Abcam  Beyotime Technology |
| Primary antibodies: |  |  |
| rabbit anti-rat CD68 | Ab283654 | Abcam |
| rabbit anti-rat CD163 | Ab82422 | Abcam |
| rabbit anti-rat C-Caspase3 | 9664 | Cell Signaling Technology |
| rabbit anti-rat Ki67 | Ab279653 | Abcam |

**Table S3. Sequence (5′ to 3′) of specific primers used for RT-PCR** **analysis**

| Genes | Forward Sequence | Reverse Sequence |
| --- | --- | --- |
| GAPDH | ACCACAGTCCATGCCATCAC | TCCACCACCCTGTTGCTGTA |
| CXCL1 | GCAGACAGTGGCAGGGATT | GGACACCCTTTAGCATCTTTT |
| CXCL2 | ATGCCTGACGACCCTACCA | GCCTTGCCTTTGTTCAGTATCT |
| CXCL5 | CTCAAGCTGCTCCTTTCTCG | GCGATCATTTTGGGGTTAAT |
| CXCL6 | CTTAGCTCCAAGAATTAACC | GGTCAAGACAAACATTATCC |
| CXCL10 | TCTCTCCACCTCCCTTTACCC | CTTGTCCATCACGCTGTAGT |
| CXCL12 | GAGTCAGACGCCTGAGGAAC | CCTTGGCCTGTCACCAATGA |
| CXCL16 | GGCTAGGCTCCATCAACGAA | ATTTGAGGTTGGCTTGGGCT |
| CCL2 | TTCACTGGCAAGATGATCCC | TGCTTGAGGTGGTTGTGGAA |
| CCL3 | CATGGCGCTCTGGAACGAA | TGCCGTCCATAGGAGAAGCA |
| CCL4 | CCAATAGGCTCTGACCCTCC | AAAGGCTGCTGGTCTCATAGT |
| CCL17 | ACCTTCACCTCAGCTTTTGGTACCATG | GCGTCTCCAAATGCCTCAGCGGGAAGG |
| CCL19 | CTACCAGCTCTGTGCACCTC | TCACACCGACTCTCTAGGCA |
| CCL21 | TCCTCAACTCAACCACAGCC | TAGGCTCGGTTCTTGCTTCC |
| CCR2 | TAGTCACTTGGGTGGTGGCT | TACAGCGAAACAGGGTGTGG |
| IL-1β | AGGCTTCCTTGTGCAAGTGT | TGTCGAGATGCTGCTGTGAG |
| IL-6 | CACTTCACAAGTCGGAGGCT | TCTGACAGTGCATCATCGCT |


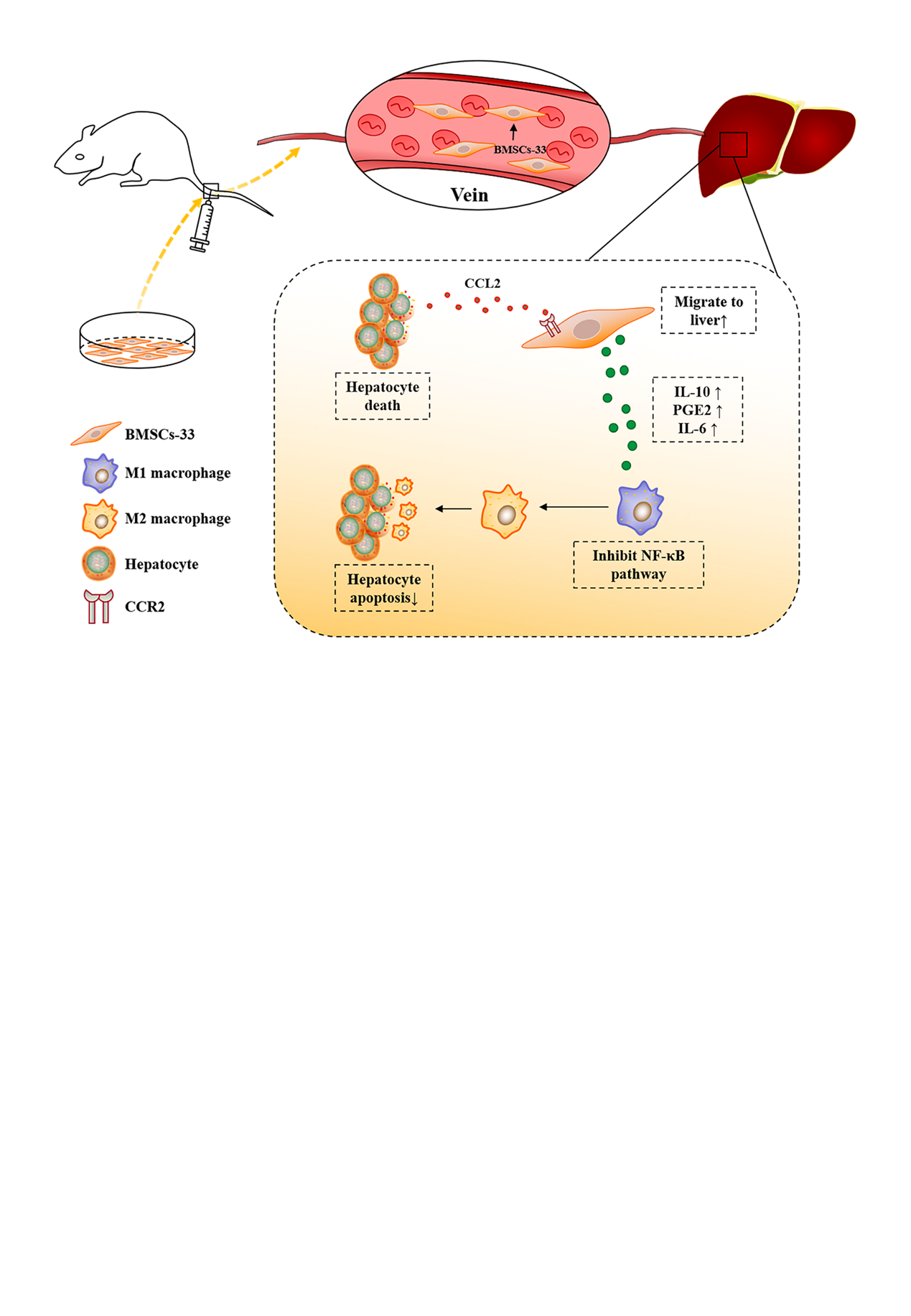


Figure S1. Schematic representation of BMSCs-33 to attenuate hepatocyte damage in rats with ALF. The expression of CCR2 in BMSCs pretreated with IL-33 increased, and it combined with CCL2 secreted by hepatocytes which could promote the migration of BMSCs. BMSCs-33 secreted IL-10, PGE2, and IL-6, which improved the inflammatory microenvironment. They inhibited the NF-κB pathway of macrophages, thereby promoting macrophage M2 polarization. Finally, M2 macrophages could reduce hepatocytes apoptosis.
